# Synthetic Polyisoprene Rubber as a Mimic of Natural Rubber: Recent Advances on Synthesis, Nanocomposites, and Applications †

**DOI:** 10.3390/polym15204074

**Published:** 2023-10-13

**Authors:** Jorge A. Cruz-Morales, Carina Gutiérrez-Flores, Daniel Zárate-Saldaña, Manuel Burelo, Héctor García-Ortega, Selena Gutiérrez

**Affiliations:** 1 Facultad de Química, Universidad Nacional Autónoma de México, Apartado Postal 70-360, Cuidad Universitaria, Coyoacán, Ciudad de México 04510, Mexico; jacruzmorales@comunidad.unam.mx; 2 Investigadora por México, CONAHCYT, Laboratorio Nacional de Análisis y Síntesis Ecológica (LANASE) y Escuela de Desarrollo Sustentable de la Universidad Autónoma de Guerrero (UAGro), Carretera Acapulco-Zihuatanejo Km 106 +900. Col. Las Tunas, Tecpan de Galeana 40900, Guerrero, Mexico; carina.gutierrez@conahcyt.mx; 3 Departamento de Química, Instituto de Educación Media Superior de la Ciudad de México, Plantel Melchor Ocampo, Calle Rosario S/N Col. Santa Catarina, Azcapotzalco, Cuidad de México 02250, Mexico; daniel.zarate@iems.edu.mx; 4 Institute of Advance Materials for Sustainable Manufacturing, Tecnologico de Monterrey, Monterrey 64849, Nuevo Leon, Mexico; 5 Departamento de Química Orgánica, Facultad de Química, Universidad Nacional Autónoma de México, Ciudad de México 04510, Mexico

**Keywords:** natural rubber, synthetic polyisoprene rubber, nanocomposites, mimic of natural rubber

## Abstract

Up to now, rubber materials have been used in a wide range of applications, from automotive parts to special-design engineering pieces, as well as in the pharmaceutical, food, electronics, and military industries, among others. Since the discovery of the vulcanization of natural rubber (NR) in 1838, the continuous demand for this material has intensified the quest for a synthetic substitute with similar properties. In this regard, synthetic polyisoprene rubber (IR) emerged as an attractive alternative. However, despite the efforts made, some properties of natural rubber have been difficult to match (i.e., superior mechanical properties) due not only to its high content of *cis*-1,4-polyisoprene but also because its structure is considered a naturally occurring nanocomposite. In this sense, cutting-edge research has proposed the synthesis of nanocomposites with synthetic rubber, obtaining the same properties as natural rubber. This review focuses on the synthesis, structure, and properties of natural and synthetic rubber, with a special interest in the synthesis of IR nanocomposites, giving the reader a comprehensive reference on how to achieve a mimic of NR.

## 1. Introduction

Natural rubber (NR) is a well-known biopolymer used for almost eight centuries. During the Second World War, a substantial increase in its demand was registered, causing several synthesis pathways to be investigated to develop a synthetic substitute. In the 1950’s, polyisoprene synthetic rubber (IR) was successfully obtained using petroleum derivatives as starting materials in the presence of Ziegler–Natta stereospecific catalysts. 

Currently, polyisoprene rubber (IR) can be obtained synthetically with different conformations (*cis*-1,4-; *trans*-1,4-; 1,2-, and 3,4-) and stereoregular controlled (isotactic, syndiotactic, and atactic), with *cis*-1,4-polyisoprene (˃98%) being the most produced and commercialized due to its elastomeric properties like high resilience and abrasion resistance, which could lead to a wide range of applications.

It is well known that some of the properties of IR are more or less similar to those of NR, with a small difference in stereoregularity. Typically, IR is composed of between 95 and 98% of *cis*-1,4-PI, compared with almost 100% of NR. Previously, it was believed that this apparently “small” difference in the stereoregularity results in the difference between NR and IR, specifically the mechanical properties [1]. Breakthroughs made in the last two decades allowed prepared IR with a *cis*-1,4-PI unit content of 99% or greater, employing a Gd-based catalyst, i.e., synthetic rubber with the same *cis*-1,4-PI unit content as the NR [2]. Unexpectedly, this synthetic rubber (*cis*-1,4-PI > 99%) exhibited inferior mechanical properties compared with those of NR, even though both polymers had the same stereoregularity [3]. As seen, the difference in the physical properties between IR and NR is attributable to a lack of understanding of the structure of NR. To our delight, it was discovered that NR is a naturally occurring nanocomposite with proteins and lipids. In order to prove this postulate, a nanostructured material consisting of NR and non-rubber components was prepared, and it was demonstrated that the mechanical properties increased dramatically when the number of non-rubber components also increased. For this consideration, a nanocompound IR with identical tensile strength to that of NR was successfully prepared [4,5].

It is important to note that, impacted by SARS-CoV-2, global economies suffered setbacks, and NR production contracted more than IR production due to a labor shortage, among other factors. This fact highlights the importance of synthetic rubber, and now, thanks to the study and preparation of nanocomposites, it is known that synthetic polyisoprene rubber (IR) can mimic natural rubber (IR). 

Although there is a long road of study on synthetic polyisoprene-based nanocomposites, there is a significant advance in the field of reinforcement additives and methodologies of synthesis, culminating in their application in the fields of tire engineering, automotive components, sporting goods, medical devices, human-tissue-mimicking materials, electrical insulating materials, shape-memory materials, and more recently in healthcare and the environment.

The primary goal of this review is to give a better understanding of the developments in synthetic polyisoprene rubber-based nanocomposites, considering the different structures, isomers, properties, production, and synthesis of IR, as well as the different methods of preparation of IR nanocomposites and their applications. 

## 2. Polyisoprene Rubber

### 2.1. Polyisoprene Synthetic (IR) and Natural Rubber (NR)

Polyisoprene exists in nature (natural rubber) but can also be produced synthetically (isoprene rubber). Natural rubber (NR) is an old biopolymer used by ancient Mesoamerican people (1600–1200 B.C.), who discovered the advantages of crosslinking it with the juice of *Ipomoea alba* L. [6]. NR is produced by more than 2000 plant species [7], most of them belonging to the Euphorbiaceae or Compositaceae families like the *Hevea brasiliensis* (willd. Ex A. Juss.) Müll. Arg. and the *Castilla elastica* Sessé ex Cerv. trees, which synthesize polyisoprenes with a *cis*-1,4 configuration; in the same way, the *Parthenium argentatum* A. Gray shrub or guayule, which occurs in the south of the United States and north of Mexico.

In contrast, only a few species, like *Palaquium gutta* (Hook.) Burck and *Manilkara bidentata* (A. DC.) A. Chev. trees, also called gutta-percha and balata [8], respectively, synthesize the isomer with conformation *trans*-1,4. According to reports, the *Manilkara zapota* or chicle tree produces a 1/4 mixture of *cis/trans* polyisoprene [9] (Ch. 29–59, [10]). In spite of the wide diversity of rubber species, *Hevea brasiliensis* is the main industrial source of NR (99%), while guayule is marketed as “non-allergenic NR” (1%) [8,11]. Thus, NR is obtained from the tree *Hevea brasiliensis* as latex, which contains about (wt/wt) 30.0–35.0% rubber, 1.0–1.8% protein, 1.0–2.0% carbohydrates, 0.4–1.1% neutral lipids, 0.5–0.6% polar lipids, 0.4–0.6% inorganic components, 0.4% amino acids, amides, and 50.0–70.0% water [8]. It is important to note that NR is obtained by coagulation of latex and has been industrially used for about 200 years now, after the discovery of vulcanization in 1838 by Charles Goodyear.

On the other hand, synthetic rubber was created in response to the strategic importance of rubber during World War II. Synthetic rubber is made of raw materials derived from petroleum. Synthetic rubbers were produced at the beginning of the last century (polybutadiene, styrene–butadiene rubber), but polyisoprene rubber (IR) was synthesized after 1950 with the development of Ziegler–Natta stereospecific catalysts [11]. Currently, IR can be obtained with different conformations (*cis*-1,4-; *trans*-1,4-; 1,2-; and 3,4-) and microstructures depending on the polymerization conditions and the catalysts used. Even the isoprene can be converted to a high *cis*-1,4-polyisoprene with physical properties similar to those of NR, and thanks to nanocompounds (IR nanocompounds), the IR can be a true mimic of NR. Thus, it is only required that isoprene be available at a low cost for polyisoprene synthetic rubber (IR) to become commercially competitive with the NR. This is discussed in detail in the next sections.

### 2.2. Structure and Isomers

**Figure 1:**
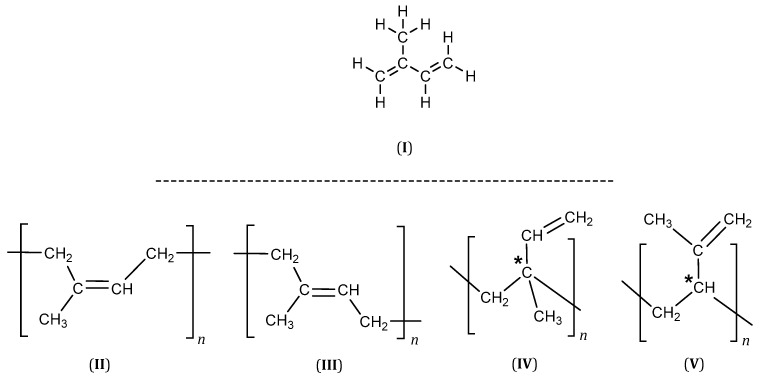
Polyisoprene structures (* an asymmetric carbon).

2-Methyl-1,3-butadiene better known as isoprene (I), is a monomer with conjugated double bonds in its structure, and its homopolymerization can lead to several polymers (Figure 1). The 1,4 addition can produce *cis*-1,4- or *trans*-1,4-polyisoprene (II, III), while the 1,2- (IV) and 3,4-additions (V) yield structures with an asymmetric carbon (marked by an asterisk), which will result in an *R* or *S* configuration (Figure 1).

**Figure 2:**
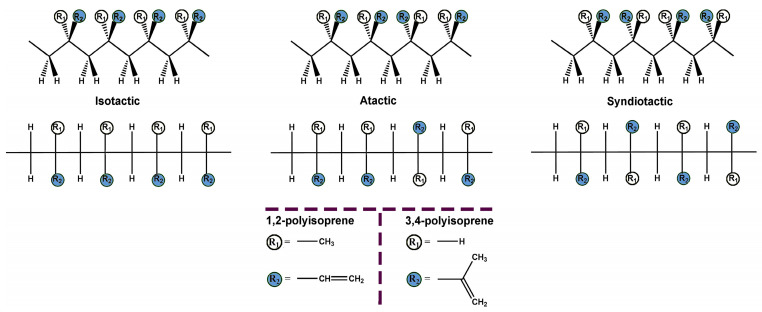
Tacticity of the polyisoprene rubber.

Thus, the arrangement of the *R* and *S* configurations along the polymer chain gives rise to diastereoisomers. Although many sequences are possible, only three simple arrangements are commonly differentiated in polymers: isotactic, atactic, and syndiotactic [12]. In isotactic polymers, all the monomer units have the same configuration (either *R* or *S*) (VI). The atactic polymer shows a random configuration (VII), while the syndiotactic polymer has a chain composed of alternating configurations (VIII). Figure 2 depicts the tacticity of the polyisoprene rubber.

**Figure 3:**
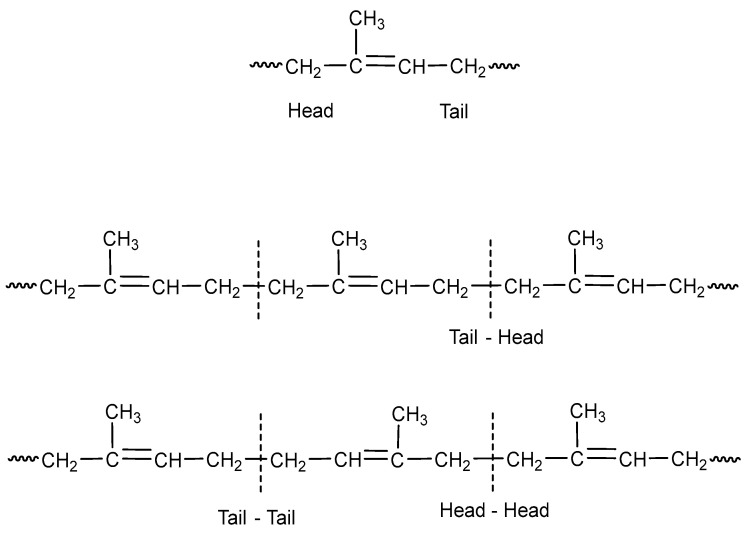
Microstructures of the polyisoprene synthetic rubber (IR).

The eight basic structures of the polyisoprene shown in Figure 1 and Figure 2 are further complicated since the microstructures have important variations. In this regard, monomer units can be linked in head-to-tail, head-to-head, or tail-to-tail arrangements, as shown in Figure 3.

As can be seen, the different arrangements and configurations discussed previously give rise to eight possible polyisoprene isomers. However, from these, the stereospecific polymerization of isoprene has permitted the synthesizing of the following highly stereoregular polymers: *cis*-1,4-; *trans*-1,4-; 3,4-isotactic; and 3,4-syndiotactic (Figure 2) [13].

High *cis*-1,4-polyisoprene (98%) [14,15] with predominantly head-tail arrangements can be obtained with titanium [16,17] or neodymium-based catalysts [18,19], when a catalyst based on gadolinium (Gd) is employed the IR obtained (99.99%) is able to mimic the *cis-* content NR (>99%) [16]. High *trans*-1,4-polyisoprene (TPI) (98%), with a structure very close to that found in natural gutta-percha (99%) and balata (99%) [12], is synthesized using titanium [20,21] or vanadium catalysts [20,22]. 3,4-syndiotactic polyisoprene was obtained with iron catalysts (80–93%) [23,24,25] and with rare earth catalysts by living polymerization [26]. The isotactic isomer (99%) is synthesized in the presence of cationic rare earth metal alkyl species resulting from a binuclear precursor [27], while an atactic 3,4-polyisoprene (90%) is obtained with the chromium system [28]. None of the three possible 1,2-polyisoprene isomers (syndiotactic, isotactic, and atactic) has been synthesized with a high yield. 

### 2.3. Properties

As described above, natural and synthetic rubbers differ in their microstructure. NR exhibits almost entirely the *cis*-1,4-polymer, whereas IR is a blend of *cis*-1,4-; *trans*-1,4-; 1,2-; and 3,4- conformations; and several microstructures: head-head, head-tail, and tail-tail (see Table 1). It is well known that an increase in *cis*-1,4 usually lowers the glass transition temperature, increases the crystallinity, and improves the mechanical strength. Previously, it was believed that the IR with a *cis*-1,4-isoprene unit content of 99.9% or greater would have the same properties as the NR. However, it has been shown that not only the *cis* content defines the properties of such material. It was recently established that NR is a naturally occurring nanocomposite formed by proteins and lipids, and precisely the nanomatrix structure gives it its extraordinary properties [4,5]. Therefore, the tensile strength and tear resistance of IR are usually somewhat lower than those of NR. However, IR nanocomposites can fully mimic the mechanical properties of NR [4], which is fundamental for specific applications. In spite of differences in mechanical properties, IR can replace its naturally occurring counterpart [29] in most industrial applications. IR high *cis*-1,4 (98%, predominantly head-tail linkages) exhibits many good properties, such as NR (high resilience, strength, and abrasion resistance), and both can be used with water, polar organic solvents (organic acids, alcohols, ketones), and some dilute acids and alkalis. However, other elastomers, such as EPDM, are preferable for these applications. By contrast, both NR and IR are attacked by non-polar solvents, fuels, and petroleum-based oils, as are the other diene-based elastomers. Furthermore, these rubbers are susceptible to attack by ozone due to the presence of double bonds in the main polymer chain, which are prone to thermal and oxidative degradation. The degradation generally occurs through chain scission and causes a drop in the mechanical properties [30].

### 2.4. Productions

The Asia–Pacific region is the unrivaled leader in terms of both natural (NR) and synthetic rubber (SR) production. In 2022, worldwide rubber production was estimated at 29.6 million tons (MMT), establishing a ratio of natural rubber to synthetic rubber of NR/SR = 49/51 [31]. Impacted by SAR-CoV-2, global economies suffered setbacks, causing world total rubber production to drop by 5.7% in 2020, with NR production (5.1%) contracting more than SR production (4.5%) due to labor shortages, among other factors [32,33]. Fortunately, during the last two years, such a trend has changed, experiencing an increase of 7.3% (in 2021) and 0.5% (in 2022) [31]. Regarding NR, around 88% is produced in the Asia–Pacific region, even though rubber trees are native to America. Regions of Europe, Africa, and the Middle East represent 9%, while North, Central, and South America only account for just less than 3% of the global NR production [33]. On the other hand, SR comprises the polymers known as large-volume production elastomers: styrene-butadiene (SBR), polybutadiene (BR), and ethylene-propylene (EPDM) rubbers, as well as *cis*-1,4-polyisoprene (IR), isobutylene-isoprene (IIR), neoprene (CR), and acrylonitrile butadiene (NBR), quite important in terms of quantity used worldwide. The rest of the SR is called specialty rubber. These are produced in small quantities but are very important for their applications. Currently, the SR industry is based primarily on the polymers and copolymers of butadiene and styrene; however, polyisoprene and copolymers containing isoprene should not be overlooked. In 2022, world SR output reached 14.9 MMT [31], where IR represented almost 5% of the market [34]. As mentioned above, SR production dropped during COVID-19; nonetheless, as the pandemic eases, global SR production rebounds steadily and will hopefully reach 17.69 MMT by 2027 [35].

About 95% of isoprene production is used to produce *cis*-1,4-polyisoprene (IR), usually known as the synthetic version of NR [36]. Table 1 shows the many types of IR manufactured industrially.

**Table 1:** Commercial synthetic polyisoprenes (IR).

	Manufacturer	Trade Names	Cat ^a^	*Cis/Trans* Content	Mooney Viscosity ^b^	Special Features	Ref.
1	*Hevea brasiliensis*	NR	N/A	99 *cis*	112	General purpose rubber	[37]
2	Goodyear Tire and Rubber, Akron, Ohio USA	Natsyn 2200	Ti	98 *cis*	80	Production of tires and other rubber products	[15]
3	Goodyear Tire and Rubber, Akron, Ohio, USA	Natsyn 2100	Ti	98 *cis*	60	General purpose rubber	[15]
4	Kraton Polymers, Houston, Texas, USA	Cariflex IR 307	Li	91 *cis*	N/A	Sensitive applications such as food contact, and pharmaceuticals, and adhesives	[38]
5	Kraton Polymers, Houston, Texas, USA	Cariflex IR 310	Li	91 *cis*	40–53	Sensitive applications such as food contact, pharmaceuticals, and adhesives	[38]
6	JCS Synthez-Kauchuk, Bashkortostan, Sterlitamak, Russia	SKI-3S	Ti	98 *cis*	72–84	Medical articles, Pharmaceutical stoppers, gaskets, hoses, and transportation belts	[39]
7	JCS Synthez-Kauchuk, Bashkortostan, Sterlitamak, Russia	SKI-5PM group II	N/A	N/A	66	Pharmaceutical application	[39]
8	JCS Synthez- Kauchuk, Bashkortostan, Sterlitamak, Russia	SKI-5PM group I	N/A	N/A	78	Pharmaceutical application	[39]
9	Zeon, Chiyoda, Tokyo, Japan	Nippol 2200	Ti	98 *cis*	82	General purpose rubber	[40]
10	Zeon, Chiyoda, Tokyo, Japan	Nippol 2200 L	Ti	98 *cis*	70	Medical articles	[40]
11	Versalis Eni, Milan, Lombardy, Italy	Europrene IP 80	Li	N/A	72	Medical articles	[41]
12	Versalis Eni, Milan, Lombardy, Italy	Europrene SOL T 9133 ^c^	Li	N/A	N/A	Hot-melt pressure-sensitive adhesives for labels or high-tack tapes	[41]
13	Sinopec, Pekin, China	SIS ^d^	Li	N/A	N/A	Adhesives, plastic, and asphalt modifications	[42]
15	Rimpex, Xiamen, Fujian, China	TPI-I	V/Ti	97 *trans*	20	Used as dispersion additives in granulation	[43]
16	Rimpex, Xiamen, Fujian, China	TPI-II	V/Ti	97 *trans*	20~40	Good machining performance, generally used in medical materials	[43]
17	Rimpex, Xiamen, Fujian, China	TPI-III	V/Ti	97 *trans*	40~60	Used in rubber products and shape-memory materials	[43]
18	Rimpex, Xiamen, Fujian, China	TPI-IV	V/Ti	97 *trans*	60~80	Used in tires and shock absorption products	[43]

^a^ Metal-based catalyst. ^b^ Mooney Viscosity ML (1 + 4) at 100 °C. ^c^ Linear block copolymer based on styrene and isoprene (SIS), where styrene content is 16 wt %. ^d^ Copolymer based on styrene and isoprene (SIS), where content is 14–16 wt %. N/A: not applicable or not available.

### 2.5. General Applications

*cis*-1,4-IR is used in a wide variety of industries and products requiring low water swell, high tensile strength, and good resilience; for instance, in rubber bands, belting, pacifiers/baby bottle nipples, conveyor belts, rubber thread, hose, tires (bicycles, aircraft, auto tires), packings, seals, motor mounts, shock absorber bushings, and other extruded and molded mechanical goods, pipe gaskets, footwear, sponges, and sporting goods [12]. Isoprene from NR or IR is also used like liquid rubber with high value-added, such as plasticizers, adhesives, functionalized liquid rubber for the synthesis of diols, macrodiols, polyols, esters, the synthesis of polymers by polycondensation reaction, and new materials with bio-based resources [44,45,46,47].

On the other hand, recent reports have shown that [48] certain substances found in natural rubber, such as protein and protein derivatives, cause irritation and allergies in the human body, so the demand for products made from alternative materials is on the rise. Thus, synthetic polyisoprene rubber has emerged as an ideal substitute for the manufacture of membranes, diaphragms, blood pressure cuff coils, seals, covers, tubes, medical gloves, condoms, different parts of medical and dental equipment, and innovative medical applications [49].

*trans*-isomer of polyisoprene has only limited commercial applications in non-elastomeric applications, such as covers for golf balls, material for orthopedic splits, electrical insulating materials, and shape-memory materials. Blends of TPI with NR, SBR, and BR have excellent processability and mechanical properties; thus, such blends can be used for high-performance tires [20].

Nowadays, a significant growth in the applications of synthetic rubber (IR) has been reported. Such applications are related to obtaining nanocomposites by adding a reinforcing material, on a nanometric scale, to the synthetic rubber matrix. IR nanocomposites have shown an increase in their properties and become more versatile, to the point of mimicking the mechanical properties of NR.

## 3. Preparation of Synthetic Polyisoprene

### 3.1. General Aspects

One of the most important polymeric materials in polymer science is polyisoprene. Over the years, research and development in the synthesis of this compound have become keystones in the rubber and tire industries. Prepared from the isoprene monomer, Bouchardat reported in 1879 the first production of a “synthetic rubbery material” using hydrochloric acid. This work quickly triggered research for a new synthetic route capable of transforming isoprene into a useful rubber [29]. 

In the mid-1950s, the development of catalyst systems allowed the polymerization of isoprene into a *cis*-1,4-polymer. One of those catalysts was a product of the intensive research of Professor Karl Ziegler, who proved that ethylene could be polymerized to a high-molecular-weight polymer in the presence of triethyl aluminum and titanium tetrachloride (Al-Ti) catalysts using mild temperature and pressure conditions. In this respect, Samuel E. Horne and co-workers successfully achieved the conversion of isoprene into high *cis*-1,4-polyisoprene (98%) employing an Al–Ti catalyst [12,50,51]. Other catalysts based on alkali metals were developed by the Firestone Tire and Rubber research team, which reported the obtention of polyisoprene with a content above 90% of 1,4 units using lithium catalysts [52].

The capability of synthesizing high *cis*-1,4-polyisoprene increased the search for an economical isoprene source and revealed several issues related to stereoregular solution polymerization. By 1967, commercial-scale production of *cis*-polyisoprene had started in the U.S. using catalysts based on trialkylaluminium-titanium tetrachloride [53].

### 3.2. Polymerization of Isoprene

The polymerization of isoprene can give four isomers of polyisoprene: *cis*-1,4-polyisoprene, *trans*-1,4-polyisoprene, 1,2-polyisporene, and 3,4-polyisoprene (in agreement with Figure 1). In practice, synthetic polyisoprene materials are not totally regular; for instance, they are structures that contain different portions of their isomers. Great interest has been focused on synthetic routes with high *cis*-1,4-polyisoprene content (above 90%) since the demand in the application fields keeps growing. Despite catalyst systems based on alkyl-lithium being able to produce as much as 98% of *cis*-1,4-polyisoprene in ideal conditions, commercially they produce 91% of *cis*-1,4- and 9% of 3,4-polyisoprene (this catalytic system is only used by Kraton company to synthesize isoprene rubber under the name of Cariflex^®^) [12,29,50,54].

Another fascinating system that allows the conversion of isoprene into high *cis*-1,4-polyisoprene is the Ziegler–Natta catalytic system, which uses a titanium halide, producing 98% of *cis*-1,4- and 2% of 3,4-polyisoprene units. The Goodyear Tire and Rubber Company began commercial production of *cis*-1,4-polyisoprene at Beaumont, Texas, with a catalyst derived from trialkylaluminum and titanium tetrachloride. Most of the commercial processes for the obtention of *cis*-1,4-polyisoprene employ one of the two catalytic systems mentioned above (Li- or Ti-based catalysts). The polymerization of isoprene is carried out using dry monomers in the absence of impurities (such as acetylene, hydrocarbons, sulfur, and nitrogen-containing compounds), air, and moisture. The presence of low levels of each may be not only detrimental to polymer conversion but can also influence the physical properties of the resulting polymer [55].

#### 3.2.1. Polymerization of Isoprene Using a Ziegler–Natta Type Catalyst System

Shortly after Professor Ziegler discovered that the product between the reaction of alkylaluminum with titanium tetrachloride (Al-Ti) polymerized ethylene at low pressure, Samuel Horne noticed that this catalyst, also called Ziegler catalysts, was capable of polymerizing isoprene in a stereospecific fashion to obtain high *cis*-1,4-polyisoprene (near to 98%) [53,54,55].

Regarding the nature of the catalyst, typical alkylaluminum compounds used in catalyst synthesis are: triethylaluminum, tri-*n*-propylaluminum, tri-*n*-butylaluminum, triisobutylaluminum, or triisohexylaluminum. The reaction between alkylaluminum and titanium tetrachloride in equimolar amounts at room temperature in a hydrocarbon solvent gives a heterogeneous system consisting of a brown solid precipitated in a yellowish solvent. The precipitated solid is mostly β-crystalline titanium trichloride with a small amount of aluminum. This catalytic system gives polymers that are prevalent in *cis*-1,4-polyisoprene (>90%) in high yields [55,56]. Other crystalline forms of titanium trichloride (α, γ, and δ) do not give *cis*-1,4-polyisoprene; instead, α-crystalline titanium trichloride and triethylaluminum give a low yield of polyisoprene with 85–90% of *trans*-1,4 units [54,55,56,57]. It is worth mentioning that high *cis*-1,4-polyisoprene (98% *cis*-1,4 and 2% 3,4 structure) can be achieved when the molar ratio of alkylaluminum and titanium tetrachloride (Al/Ti) is equal to 1, whereas small ratios (0.7–0.8) can decrease yields, and a rubbery material mixed with increasing amounts of a leathery-resinous can be obtained [58].

Due to the heterogeneity of the Al–Ti reactions, it is obvious that considerable art is involved in making optimum catalysts for isoprene polymerization. There are two components, alkylaluminum and titanium tetrachloride, that are usually diluted in an inert solvent and can react in the absence of the monomer to produce a preformed catalyst or be mixed in situ in the presence of the monomer. Preformed titanium tetrachloride-triisobutylaluminum catalyst systems are more reactive and provide more reproducible polymerizations than catalysts prepared in situ. On the other hand, aged catalysts give polymers nearly free of low molecular-weight oils, but the best catalytic performance and polymer properties are obtained with catalysts performed below 20 °C by the gradual addition of the alkylaluminum to the titanium tetrachloride or by mixing the aluminum and compounds at a constant Al/Ti ratio of 0.9 to 1.0. The polyisoprene microstructure is 97–98% *cis*-1,4- [59]. 

Suitable solvents for the polymerization of isoprene are aliphatic and aromatic hydrocarbons. Low-boiling aliphatic solvents (butane, pentane, and hexane) have been preferred for large-scale commercial production since their high vapor pressures permit reflux cooling for temperature control. An important issue in the polymerization of isoprene is gel formation. In aliphatic solvents, the gel content is usually between 20 and 35%, but in aromatic solvents, it is much lower. Gel content is relatively independent of polymer conversion and catalyst concentration [54,60]. 

*trans*-1,4-Polyisoprene (TPI) can be obtained by a vanadium cocatalyst. The trialkylaluminum–vanadium trichloride catalyst system with a molar ratio of 2.0–3.5/1, in heptane at room temperature gives high TPI with similar properties to gutta-percha rubber [61,62]. Another catalytic system with a potential commercial application is vanadium trichloride–triethylaluminum, VCl_3_-(C_2_H_5_)_3_Al, which gives 98% of *trans*-1,4-polyisoprene and 2% of 3,4-polyisoprene [63].

#### 3.2.2. Polymerization of Isoprene Using Rare-Earth Compounds

The polymerization of isoprene using rare earth compounds—trialkylaluminum halide catalysts—was initially reported in China; in 1974, the data showed 95% of *cis*-1,4-polyisoprene with a high molecular weight [64]. The general formula for this kind of catalyst is Ln(naph)_3_-(*i*-C_4_H_9_)_3_Al-(C_2_H_5_)_3_Al_2_Cl_3_, where Ln is a rare earth metal ion and naph is naphthenate. The catalyst of the highest activity is that constituted by neodymium naphthenate, Nd(naph)_3_, which is capable of producing 96% of polyisoprene with 95% of *cis*-1,4- and 5% of 3,4- microstructure. On the other hand, praseodymium naphthenate, Pr(naph)_3_, produces a 91% conversion to polyisoprene with 93.9% of *cis*-1,4-; 0.6% *trans*-1,4-; and 5.5% of 3,4-polyisoprene microstructure.

In general, catalysts made of lanthanum, cerium, praseodymium, and neodymium produce about 95% of *cis*-1,4-polyisoprene [65]. The remaining lanthanide elements show slightly higher *cis*-1,4-polyisoprene content but lower catalytic activity. Another type of catalyst capable of polymerizing isoprene are binary systems composed of tetrahydrofuran adducts of lanthanide chlorides and triethylaluminium, NdCl_3_∙2THF-(C_2_H_5_)_3_Al. These systems give 95% *cis*-1,4- and 5% 3,4- microstructures in toluene at 30 °C, with a high conversion above 94%. Reactivity order in rare-earth elements is Nd > Pr > Gd.

#### 3.2.3. Polymerization of Isoprene Using Alkali Metals

The recognition of alkali metals and organo-alkali as initiators in the polymerization of the conjugated dienes was made by Mattews, Strange, and Harries in the mid-1950s [12,65,66,67]. The polymerization process of isoprene using lithium as an initiator produces a characteristic IR comparable to NR, containing over 90% of *cis*-1,4-polyisoprene. Lithium-dependent initiators are unique compared with other alkali metals since they can produce polyisoprenes containing 1,2-, 3,4-, and *trans*-1,4-polyisoprene. It is remarkable how important the role of the solvent is for the polymer structure in the polymerization process. For example, when polymerization was carried out with *n*-butyllithium in heptane, the major product obtained was *cis*-1,4-polyisoprene (above 90% of products), whereas using another solvent such as diethylether, 1,2-, 3,4- and *trans*-1,4-polyisoprene were obtained without evidence of *cis*-1,4-polyisoprene [68]. It is worth mentioning that polymerization of isoprene using alkali metals and organo-alkali compounds is considered a heterogeneous reaction when it is carried out both in bulk and in hydrocarbon solvents. On the contrary, the homogeneous reaction takes place only in the presence of highly polar solvents. Other initiators using lithium, sodium, and potassium have been studied [69]**,** finding that the reaction product of these alkali metals with thiobenzophenone in tetrahydrofuran gives a soluble initiator, generating 68–75% of 3,4 structures and no 1,4 structures [54,70]. The reaction product of sodium, potassium, rubidium, or cesium with poly(*p*,*p*′-biphenyleneethylene) in benzene or THF is another initiator [71]. The potassium-reaction product yields 60% of *trans*-1,4-, 30% of 3,4-, and 10% of *cis*-1,4-isoprene units. In general, alkali metals have mixed polymer structures.

Crown ethers (macrocyclic polyethers) form soluble initiators of alkali metals in tetrahydrofuran and benzene. Dicyclohexyl-18-crown-6 (DCHE) gives stable solutions of sodium, potassium, rubidium, and cesium in tetrahydrofuran and benzene. These solutions rapidly initiate the polymerization of isoprene. The inconvenience associated with conventional alkali metal catalysts is avoided when the polymer is formed on the metal surface. The polymerization of isoprene with sodium metal and DCHE in benzene at 10 °C gives 32% of 1,4-; 44% of 3,4-; and 24% of 1,2-polyisoprene microstructure. The polymerization in tetrahydrofuran at −78 °C gives 12% of 1,4-; 58% of 3,4-; and 30% of 1,2-polyisoprene microstructure [72].

#### 3.2.4. Polymerization of Isoprene Using an Alfin Catalyst

The alfin catalysts are special combinations of sodium salts, one from alcohol and the other from olefin, which have unique properties as polymerizing agents for diene compounds. The most common alfin catalyst is a mixture of allyl sodium, sodium isopropoxide, and sodium chloride in molar proportions of 0.35:1.0:1.5, respectively [12,54]. When isoprene is polymerized using an alfin catalyst, a great amount of *trans*-1,4- configuration is obtained with about 20% of double bonds. The molecular weight is controlled by small amounts of 1,4-dihydrobenzene or 1,4-dihydronaphtalene; these do not affect the microstructure of the resulting polyisoprenes [73]. Such polymers are processed readily on standard rubber machinery. Compared to NR, alfin polyisoprenes exhibited lower tensile strength and initial tear strength but improved abrasion resistance. The microstructure of a typical sodium alfin polyisoprene is 27% *cis*-1,4; 52% *trans*-1,4-; 16% 3,4-; and 5% 1,2-polyisoprene [68].

#### 3.2.5. Polymerization of Isoprene Using a Metallocene Catalyst

Metallocenes are known for most metals (discovery of ferrocene in 1951 and report of its sandwich structure in 1952) and have applications in organic, polymer, and medicinal chemistry [74]. Metallocene catalysts show, in contrast to Ziegler systems, only one type of active site (single site catalysts), which produces polymers with a narrow molar mass distribution (Mw/Mn = 2), and their structure can be easily changed. They are soluble in hydrocarbons or liquid propene.

It is known that metallocenes are effective catalysts for synthesizing polybutadiene. Catalyst systems based on coordination compounds, i.e., lanthanide metallocenes with cocatalyst, are very effective for *cis*-1,4- stereospecific polymerization of butadiene. Specifically, (C_5_Me_5_)_2_Sm[(μ-Me)AlMe_2_(μ-Me)]Sm(C_5_Me_5_)/[Ph_3_C][B(C_6_F_5_)_4_]/Al*i*Bu_3_ gives a polybutadiene with high *cis*-1,4- content (>99.5%) [75]. However, this samarocene-based catalyst system does not induce the polymerization of isoprene under various conditions [2]. In this regard, it was recently found that the gadolinium analog can more efficiently catalyze the polymerization of butadiene, allowing for the synthesis of almost perfectly *cis*-1,4- regulated polybutadiene (>99.99%) [76]. As for related polyisoprene polymerization, the gadolicene-based catalyst (C_5_Me_5_)_2_Gd[(μ-Me)AlMe_2_(μ-Me)]Gd(C_5_Me_5_) alone does not induce the polymerization of isoprene. Nevertheless, adding [Ph_3_C][B(C_6_F_5_)_4_]/Al*i*Bu_3_ converts the system into a ternary catalyst system that not only initiates the polymerization of isoprene, but the selectivity of the *cis*-1,4-polyisoprene dramatically increased up to 98.7% at 0 °C and reached >99.99% at −20 °C with no evidence for the presence of a trace of *trans*-1-4- or 3,4-polyisoprene [2]. (These results have not been industrialized yet).

## 4. Nanocomposites

Nanocomposites are composites that consist of several phases where at least one of their dimensions is in the nanometer scale (10^−9^ m). Nanocomposites represent an alternative way to overcome the current limitations of microcomposites and monolithic materials, becoming a widely studied materials field with an estimated annual growth rate of 29.5% for the period of 2017–2022 [77].

The structure of nanocomposites consists of a matrix material containing the nanosized dispersed reinforcement components in the form of fibers, nanotubes, particles, and whiskers, among others. The advantage of nanofillers compared to more conventional micrometer-sized fillers is that, for the same concentration, nanofillers usually provide better properties [78]. 

The enhancement of properties of nanocomposites, such as optical, mechanical, thermal, and barrier, is one of their main advantages over other composite materials. Currently, nanocomposites offer new technology and business opportunities for many zones of industry, and in addition, some show environmentally friendly properties [79]. 

There is a wide innovative variety of applications for nanocomposites in many fields: semiconductors, catalysis, environmental remedies, energy, medicine, sunscreens, and biomaterials, mentioning some that have grown and progressed in recent decades [80,81]. For instance, it is fascinating to see blossoming in medical applications, in particular, tissue engineering. In this sense, the synthesis of nanocomposites that faithfully mimic the mechanical behavior of biological tissues, ranging from soft fat tissue to firm human skin, has been reported [82,83,84].

Recently, dramatic growth in research in the synthesis of nanocomposites has created the need for classification. Consequently, nanocomposites can be classified by the nature of the matrix into ceramic-based, metallic-based, and polymeric-based [77,80].

### 4.1. Ceramic Matrix Nanocomposites (CMNs)

Ceramic matrix nanocomposites are constituted by a matrix formed by ceramic material (oxide or non-oxide), which is reinforced with whiskers, fibers, platelets, or different types of particles, all of them on the nanometric scale. Such nanofillers improve mechanical, antimicrobial, and fire-retardant properties, as well as chemical and thermal stability, anti-corrosion, self-healing properties, elasticity, and biocompatibility, among others [85,86]. Between the most promising reinforcements, it can summarize SiC, carbon particles (e.g., nanotubes and graphene), Al_2_O_3_, ZrO_2_, orthosilicates (e.g., mullite mineral), and aluminosilicates (e.g., montmorillonite clay).

### 4.2. Metal Matrix Nanocomposites (MMNs)

Metal matrix nanocomposites are multiphase materials composed of a metal matrix with some implanted nanosized reinforcement that includes oxides (e.g., Al_2_O_3_ and SiO_2_) [87,88], carbides (e.g., SiC, B_4_C, and TiC) [89], nitrides (e.g., AlN and BN) [90], and elemental materials like carbon (e.g., nanotubes and graphene). Typical goals in the production of these materials are the improvement of properties such as tensile strength, thermal stability, Young’s modulus, and wear resistance [80].

### 4.3. Polymer Matrix Nanocomposites (PMNs)

**Figure 4:**
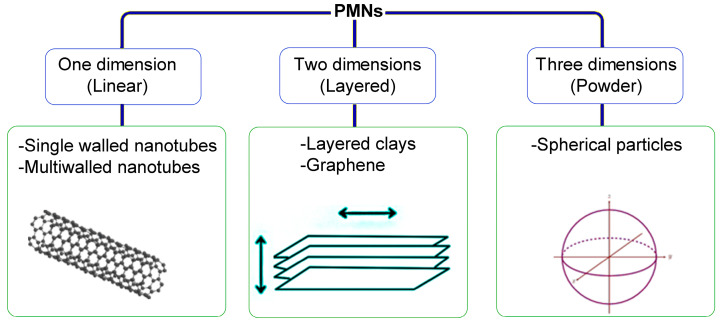
Polymer matrix nanocomposites (PMNs) classification.

These materials are composed of a continuous polymeric phase and a nanofiller as the discontinuous phase. PMNs were first discovered by the Toyota research group back in the early 1990s [91]. All types of polymers, including elastomers, thermoplastics, thermosets, and specialty polymers, have been reported to prepare PMNs. On the side of reinforcement, the used materials include metal powders (e.g., Fe) [92], crystalline structures, ceramic compounds (e.g., clays, silica, TiO_2_), carbon-based materials (e.g., fullerene, nanotubes, and graphene), biofillers (e.g., peat, wood, and lignin), and polyhedral particles. Among the characteristics achieved are high thermal stability, improved mechanical properties, and lower gas permeability. According to nanofiller geometry, PMNs can be classified as linear (one dimension), layered (two dimensions), or powder (three dimensions), as shown in Figure 4 [93].

### 4.4. Rubber Matrix Nanocomposites (RMNs)

Rubbers, either natural or synthetic in origin, also referred to as elastomers, are classified based on the monomer (e.g., polyisoprene, nitrile, styrene, and neoprene) in the macromolecular chains. Meanwhile, the properties of raw elastomers are rather unsatisfactory; they are commonly filled with small and hard particles to improve their thermal and mechanical properties, like elastic modulus or resistance to abrasion. A basic requirement for attaining optimum reinforcement is a fine dispersion of filler that results in a good adhesion polymer/filler [94].

**Figure 5:**
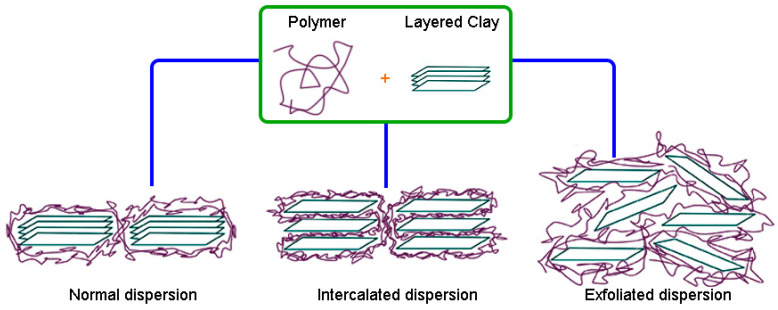
Possible dispersion of layered clay in a polymer matrix.

Carbon black and mineral fillers such as silica have been widely used in the rubber industry for the past many decades to improve properties [95]. However, due to their rather high cost and environmental impact (since carbon black is petroleum-based), much attention and interest have been focused on searching for alternative additives that are cheaper and more eco-friendly to reduce environmental impact. Compounds like layered clays such as montmorillonite, kaolinite, and bentonite and their respective organophilized modifications are probable candidates that could supplant the traditional rubber additives for nanocomposite production. The main challenges to be solved in this field in the future are the complete exfoliation and homogeneous dispersion of these clay reinforcements to obtain RMNs, as shown in Figure 5 [96].

Beyond clays, in recent years, two kinds of fillers have been widely studied and attracted a lot of attention: biofillers and polyhedral particles.

**Figure 6:**
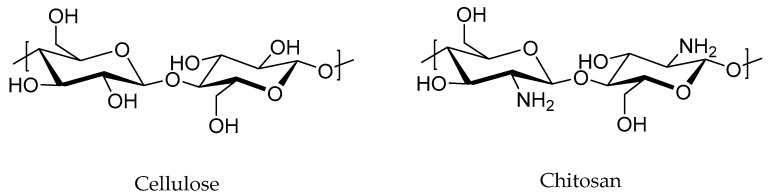
Example of some biofillers used nowadays.

Biofillers, such as natural-derived nanofillers such as cellulose fibers from wood, chitosan, starch, and lignin (Figure 6), have been recently explored for effective incorporation in diverse rubber matrices. It is worth noting that the development of RMNs using bionanofillers is still in its early stages of research due to the difficulty in the extraction of nanofillers from natural sources [97].

**Figure 7:**
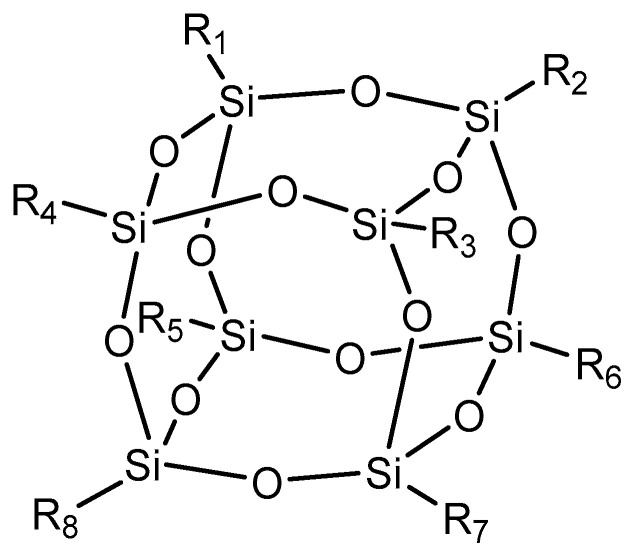
Silsesquioxane, an example of polyhedral reinforcement.

In the case of polyhedral fillers, specifically polyhedral oligomeric silsesquioxane (POSS) (Figure 7), this is considered a breakthrough in nanotechnology. The incorporation of this filler can provide enhanced properties while maintaining processability. POSS promotes preferential chemical bond formation between rubber and its surface, maintaining its molecular volume and providing a significant extent of mechanical reinforcement, showing predefined size and functionality. Additionally, this filler offers compatibility with a wide variety of rubbers, increasing its potential application in chemical technology and covering the automobile, medical, and electronic industries in the near future [98,99,100].

In the field of RMNs, there are several challenges to face. For instance, the complete exfoliation and uniform dispersion of nanofillers in the rubber matrix, as well as an efficient surfactant, must be designed for excellent dispersion. In the case of clay-filled rubber nanocomposites, the extent of exfoliation/intercalation has not been quantified yet. The orientation of nanoplatelets in the rubber matrix by special extrusion is also a major challenge. The in-situ monitoring of the flow of rubber nanocomposites during processing needs a lot of attention [101].

## 5. Preparation of Synthetic Polyisoprene Rubber Nanocomposites (IR Nanocomposites)

It is reported that natural rubber presents properties of nanocomposite of naturally occurring nanocomposites due to the content of non-rubber components such as proteins and lipids, which improves its thermal and mechanical properties compared to IR synthetic rubber. The synthetic rubber is prepared by chemically attaching nanoparticles onto microparticles of synthetic *cis*-1,4-polyisoprene dispersed in water as a colloidal dispersion, followed by drying to form an “island-nano matrix structure” similar to that of NR [4].

In general, the nanocomposites of both elastomers can be synthesized using the same methods. Which can be divided into five major groups according to the processing techniques [102,103,104,105,106,107]:

- In situ polymerization;
- Solution blending (solution intercalation);
- Melt compounding (melt intercalation);
- Sol–gel method;
- Latex compounding.

### 5.1. Methods for the Synthesis of Nanocompounds

#### 5.1.1. In Situ Polymerization

**Figure 8:**
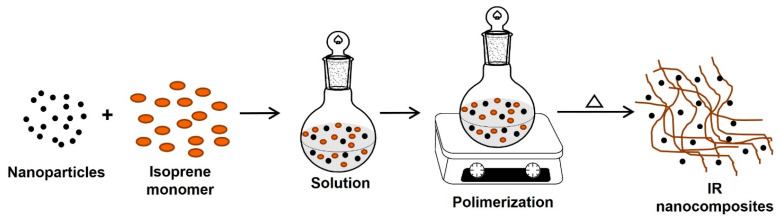
Synthesis of IR nanocomposites by in situ polymerization.

In this method, the nanoparticles (nanofillers) are swollen into the monomer solution (or liquid monomer), leading to a homogeneous mixture that can be polymerized by incorporating a curing agent or catalyst or increasing the temperature. Thus, the formation of rubber can occur between and around the nanoparticles; consequently, a good dispersion of the nanofillers in the rubber matrix is obtained. If the nanofiller is clay, polymerizing the monomer in the interlaminar space causes the “break” of the laminar structure, thus achieving good dispersion of the clay sheets in the rubber to obtain the IR or NR intercalated/exfoliated nanocomposites (Figure 8) [102,103].

#### 5.1.2. Solution Blending

**Figure 9:**
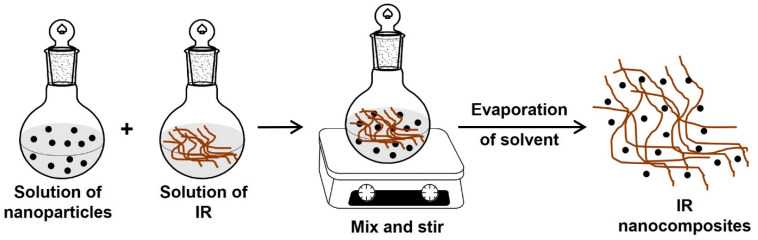
Synthesis of IR nanocomposites by solution blending (solution intercalation).

The obtention of IR nanocomposites by solution blending consists of independently dissolving the rubber and nanoparticles in a suitable solvent (organic solvent). Then, the solutions are mixed and stirred for some time, and finally, the solvent is evaporated (Figure 9) [102,103,105].

This method is indicated for the intercalation of polymers with low or no polarity, such as polyisoprene rubber, in nonpolar solvents like toluene, chloroform, or tetrahydrofuran, allowing the production of thin films with the polymer chains and the nanoparticles or clay sheets oriented. Unfortunately, the solution blending method is not eco-friendly and cost-effective due to using an excess amount of organic solvents [103,105].

#### 5.1.3. Melt Compounding (Melt Intercalation)

**Figure 10:**
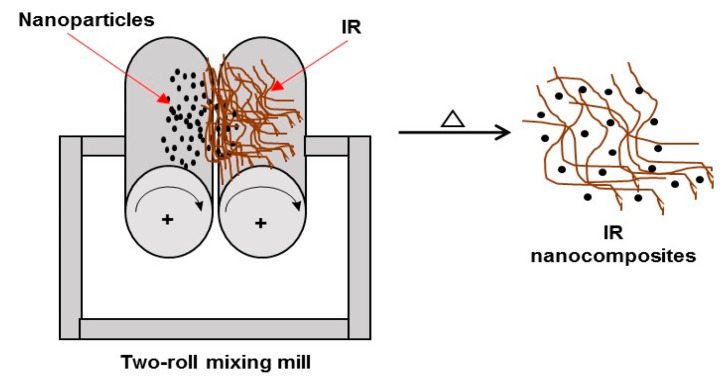
Synthesis of IR nanocomposites by melt compounding (melt intercalation).

The formation of nanocompounds by melt compounding involves physical mixing at high temperatures, usually carried out in an extruder, a roller mixer, or open mills. The process begins by loading the selected equipment with the rubber and the nanoparticles (nanofillers) mixed at high temperatures. Then, the rubber and the nanoparticles produce a homogeneous mixture in the molten state, in which the rubber chains are entered into the nanoparticles to form the IR nanocomposites (Figure 10) [102,103,105].

#### 5.1.4. Sol–Gel Method 

**Figure 11:**
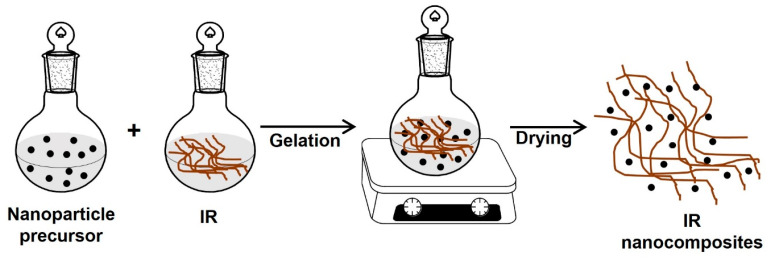
Synthesis of IR nanocomposites by the sol–gel method.

This technique allows the synthesis of the nanoparticles or sheets inside the polymer matrix. First, an aqueous or gel solution that contains the polymer and the nanoparticle precursor (nanofillers) is prepared; the gel or solution is mixed and stirred for a while; and finally, the solution is dried to obtain the IR nanocomposites (Figure 11).

The main disadvantage of this process is that the high temperatures required to assemble the reinforcement can cause the decomposition of the polymer [108,109].

#### 5.1.5. Latex Compounding

Latex compounding is a promising method for preparing rubber nanocomposites where the principal medium is the water contained in latex; for this reason, this method is exclusive to polyisoprene from NR. The latex compounding technique starts with dispersing nanoparticles in water that acts as a swelling agent owing to hydration; then, the rubber latex is added and mixed for a while to achieve a complete dispersion of nanoparticles in the solution. Subsequently, the rubber nanocomposite of the mixture is coagulated by adding formic acid, and finally, the water is removed [102,103,104].

All methods described above allow the synthesis of IR nanocomposites with a different dispersion degree. Several characterization techniques can be employed to confirm the formation of nanocomposites, evaluate their dispersion, and analyze the surface area of polymers [105,110,111,112,113,114,115,116]. For instance, scanning electron microscopy (SEM) and transmission electron microscopy (TEM) are used to see the morphology of nanocomposites, surface area and textures, the dispersion of nanoparticles in the rubber, homogeneous distribution, agglomerates, or regions with a fully exfoliated structure [105,110,111,112,113,114,115,116]. Atomic force microscopy (AFM) is used to study the agglomerations or heterogeneity of nanocomposites in rubber [105]. X-ray diffraction (XRD) or wide-angle X-ray diffraction (WAXD) to evaluate the state of dispersion of nanocomposites in the rubber diffraction peaks to relate compatibility with the matrix and crystallinity. In clays, XDR and WAXD permit analysis of the interlayer spacing. The intercalation of polymer chains is characterized by an increase in interlayer spacing, while the absence of diffraction reflects a complete clay exfoliation [111,112,113].

### 5.2. Improvements and Applications of Polyisoprene Rubber and Natural Rubber Nanocomposites

Synthetic polyisoprene rubber (IR) is produced mainly anionically and by Ziegler–Natta polymerization. IR rubber is often used in the same applications as NR, behaving during the mixing and processing stages like NR due to their chemical similarity [102,117]. While NR has up to a 99.9% *cis*-1,4- microstructure, depending on the species, IR may be as much as 98% stereoregular. Although there is a small difference in stereoregularity, the higher properties of NR are attributed to its structure, which is considered a naturally occurring nanocomposite, as mentioned above. Therefore, IR is substantially more crystallizable and exhibits inferior mechanical properties than its natural counterpart [4]. This can be seen in Table 2 (entries 1 and *2*), where the tensile strength (MPa) and elongation at break (%) values for NR and IR are 5.9/780 and 0.1/480, respectively.

In recent years, IR nanocomposites have attracted attention in academic research and the industrial field since their mechanical, thermal, barrier, and flame-retardant properties can be improved at lower loadings of fillers (<10 wt %) compared with conventional polymer composites (30–70 wt %). This represents lower manufactured costs and a significant increase in potential applications [106,118,119,120]. In this respect, IR nanocomposites are suitable materials to be widely used in many applications, such as chlorinated and isomerized rubbers for the surface coatings industry [102,121], tire engineering, automotive components, membranes, sporting goods, sports balls, golf covers, medical devices and healthcare, vibration-absorptive materials, electrical devices, electrical insulation materials, shape-memory materials, as well as the electronics and aviation industries [111,112,122,123,124,125].

To achieve the required physical-mechanical properties, rubbers must be reinforced with fillers. The beneficial effects of carbon black were discovered at the beginning of the twentieth century. However, its large-scale application was hindered by consumer resistance to the black color, for example, in the production of tires. Alternatively, silica was also used in rubber formulations at the beginning of the last century. Still, only towards the end did it become the preferred filler to allow safe driving on wet surfaces and reduce fuel consumption [122,126].

In line with the applications, Galimberti et al. reported the synthesis of nanocomposites with IR (*cis*-1,4-polyisoprene) and clays. Organoclays used in a minor amount to replace carbon black in IR, NR, and SBR-based formulations bring about a substantial decrease in raw compound viscosity and, after sulfur curing with appropriate vulcanization kinetics, promote a more linear stress-strain curve with better ultimate properties and a remarkable increase in storage modulus. In this work, the authors concluded that organoclay could become an ideal filler for rubber compounds, improving both rheological and reinforcement properties if vulcanization parameters are under control and reinforcement is stable in a wide range of temperatures [122].

**Figure 12:**
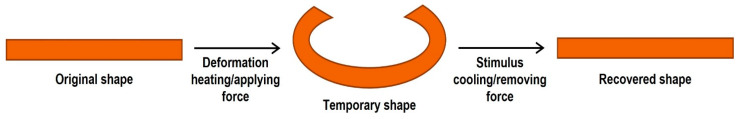
The shape memory of *trans*-1,4-polyisoprene (TPI).

Another synthetic polyisoprene rubber application with conformation TPI is in shape-memory materials, which are a class of smart materials capable of altering their state parameters under external stimuli, such as temperature, force, pH, solvent, magnetic fields, light, and electric fields, among others, and recovering to their initial states when the external stimulus ceases (Figure 12) [112,123]. TPI is a new type of synthetic rubber with shape memory; its most outstanding characteristic is its thermoplasticity and recovery ability from deformations greater than 200% [112,123,127]. Due to this characteristic, TPI is an interesting, smart material into which nanofillers can be incorporated to improve some of its mechanical, thermal, or electrical properties through TPI nanocomposite synthesis.

In this sense, Liu et al. developed functionalized core–shell nanohybrid/synthetic rubber nanocomposites with enhanced performance, with synthetic TPI as a matrix and SiO_2_ and GO (graphene oxide) as fillers. The nanocomposites were produced by melt compounding and synthesized to study the effects of the different components on their thermal and mechanical properties. SEM, TEM, and AFM confirmed that GO sheets were uniformly affixed to the SiO_2_ particles’ surfaces, finding that those nanocomposites with SiO_2_/GO (1.0 wt %) exhibited the best thermal and mechanical properties compared to all other samples; even these nanocomposites exhibited good shape memory properties [112].

Cao et al. reported in situ polymerization of isoprene using reduced graphene oxide (rGO) and carbon nanotube (CNT)-supported Ziegler–Natta catalysts. TPI nanocomposite increased crystallinity and contained well-dispersed nanofillers. The polymer nanocomposite displayed enhanced mechanical properties, thermal conductivity, and electrical conductivity. Only with 2 wt % rGO/CNT did the TPI nanocomposites show improved mechanical properties (e.g., an increase of 110% modulus at 300% strain), 65% increased thermal conductivity, and 109 times increased electrical conductivity, which leads to a high-performance multifunctional material and enables a range of applications [111]. On the other hand, it is reported that the use of graphene as a reinforcement nanocomposite in NR significantly improves the mechanical properties, such as the elastic modulus, bulk modulus, and shear modulus, in addition to the tribological properties, compared to pure NR [128]. Similarly, using CNT to prepare CNT/NR nanocomposites improves their physical and mechanical properties. However, it affects the vulcanized NR’s curing process, and additional heating is required to cure CNT/NR nanocomposites due to their higher active energy [129].

One of the major applications of rubbers is in the tire industry; it was reported that the global demand for tires reached 3.2 billion units in 2022. They are synthetic rubbers such as SBS, SBR, BR, butyl rubber (IIR), IR, and natural rubber [130]. Different rubber types are usually employed in the fabrication of tires, mainly NR and synthetic rubber blends, including IR. The incorporation of nanocomposites into tire components is driven by potentially higher overall performance, especially focused on fuel efficiency through reduced weight and energy absorption from rolling resistance (according to the so-called magic triangle performances for a tire tread: rolling resistance, traction, and wear), and more favorable economics deriving from easier processing, reduced complexity of construction, and substitution of less expensive polymers [104,119].

In this regard, using a sol–gel process, NR vulcanized for tire applications was reinforced with in situ-formed nanoscale silica particles (around 40 nm in diameter). The in situ silica filling of NR using the sol–gel method has a potential application for various rubber products exposed to severe friction and abrasive wear conditions, like tire tread and driving belts [131].

Similar behavior was found for IR matrix vulcanizates prepared with an organoclay (fluorohectorite modified by octadecylammonium with an interlayer distance of 2.24 nm), suggesting that reinforcement is related to the anisotropic nature of the aggregates, and orientation during strain significantly improved modulus and tensile strength [119,132].

Another current application is for preparing functional nanocomposites, colloidal polymers, and core–shell materials using NR latex and inorganic nanoparticles such as SiO_2_, Ag, Au, TiO_2_, and magnetic particles. Designed to improve the antimicrobial, electrical, thermal, optical, and magnetic properties of the NR-based nanocomposites, which can be used in various applications, including nanofillers, controlled-releasing materials, textiles, electronics, and coatings, among others [133]. In a study published in 2023, adding Ag nanoparticles as a filler material for the NR (a volume ratio of 1:10 Ag/NR) created interfacial polarization between the conductive metal nanoparticle and the insulating polymer, thereby improving the dielectric constant and triboelectric nanogenerator. The application of Ag/NR as a shoe insole was demonstrated to convert human footsteps into electricity to power small electronic devices, and with the presence of Ag nanoparticles, the fabricated shoe insole also exhibits antibacterial properties against Staphylococcus aureus that causes foot odor [134].

**Table 2:** Physical properties of IR, NR, and their nanocomposites with different fillers.

Entry	Rubber	Nanocomposite Filler (%)	Vulcanized	Properties				Ref.
				Tensile Strength (MPa)	Strain Max. (%)	Hardness (Shore A)	Thermal Decomposition (°C)	
1	NR	--	--	5.9	780	--	--	[4]
2	IR	--	--	0.1	480	--	--	
3	IR	nano PS (graf)	--	3.9	700	--	--	
4	NR	--	√	34.5	550	--	--	
5	IR	--	√	27	650	--	--	
6	IR	nano PS (graf)	√	35.2	600	--	--	
7	TPI	--	√	8	350	--	343	[112]
8	TPI	GO (1%)	√	20	280	--	352	
9	NR	--	√	15.1	995	--	385	[110]
10	NR	Silica SiO_2_ (4%)	√	26.3	730	--	395.4	
11	NR	--		4.25	>700	28.8	--	[135]
12	NR	MMT (10%)	√	3.6	555	20.7	--	
13	NR	OMMT (10%)	√	15.0	700	43.5	--	
14	NR	CB (10%)	√	4.93	464	30.5	--	
15	NR	--	√	15.45	592	46	374	
16	NR	AT (1%)	√	23.27	432	50	393	[136]

√ = Affirmative value. -- = Not contain or value not reported (properties). GO = Graphene oxide. MMT = Montmorillonite clay. OMMT = Organomodified montmorillonite. CB = Carbon black. AT = Attapulgite clay. Thermal decomposition (Td) is calculated by TGA.

Table 2 shows clear examples of how the preparation of nanocomposites has contributed to improving the physical properties of both natural and synthetic rubber (NR and IR), vulcanized or non-vulcanized. For instance, entries 8, 10, 13, and 16 (Table 2) indicate that the tensile strength, hardness, and thermal decomposition of nanocomposites of IR increase in relation to neat rubber (Table 2, entries 7, 9, 11, and 15; respectively), in contrast to the maximum strain, which with the increase of the filler tended to decrease. In all cases, the tensile resistance value is higher for the vulcanized rubber than for its non-vulcanized counterpart. This trend is also reflected in the corresponding nanocomposites.

Another important fact to highlight about nanocomposites is that they allow IR to mimic the outstanding properties of NR. It is well known that NR exhibits higher mechanical properties (entry 1, Table 2) than its synthetic counterpart (entry 2, Table 2). However, it has been demonstrated that the nanocomposites of IR (entry 3, Table 2) are capable of mimicking the properties of NR (entry *1*, Table 2). For instance, the tensile strength value for NR is 5.9 MPa, compared to 3.9 MPa for nanocomposites of IR. This behavior can be more clearly observed in both polymeric materials after their vulcanization. Vulcanized NR shows a tensile strength (MPa) of 34.5 (entry 4, Table 2), while the vulcanizate IR nanocomposite achieves a slightly higher value of 35.2 (entry 6, Table 2). It is important to note that neat vulcanizate IR (entry 5, Table 2) shows a tensile strength of 27.0 MPa.

Furthermore, breakthroughs made in the last two decades have demonstrated that the physical properties of synthetic rubber can be increased regardless of whether the neat rubber used in the preparation of the nanocomposite has a high *cis* or *trans* conformation (entries 6 and 8, Table 2). However, to gain a better understanding of this, new studies must be conducted to determine how exactly the *cis*/*trans* ratio influences nanomaterial properties. Another interesting variable that must be considered is the crosslinking density, because linkages between chains directly influence the physical properties. It is interesting to see how the management of all these variables can lead to a wide gamma of polymeric nanocomposites with very specific properties for diverse applications such as high-performance tires, medical devices, electrical insulating materials, shape memory materials (widely used in the auto industry, the electronics industry, the aviation industry, and medical apparatus), or human-tissue-mimicking materials, among others.

Currently, IR nanocomposites can mimic the properties of NR and thus wholly replace it, especially when this valuable resource cannot meet global demand due to extraordinary conditions, such as the pandemic due to SAR-CoV-2.

## 6. Conclusions 

IR has properties more or less similar to those of NR and can replace it in many industrial applications. However, several significant physical properties of IR are not as good as NR. In this sense, nanocomposites emerged as a response. In these, properties such as tensile strength and tear resistance increase due to the presence of a nanometer-sized reinforcing material, which is added at a low load (<10 wt.%) compared to conventional polymeric composites. This means a lower manufacturing cost and a significant increase in potential applications. 

Another fact that contributed to the synthesis of IR nanocomposites was solving the mystery of the structure of NR and thus understanding their outstanding properties. NR is a naturally occurring nanocomposite and was a bioinspiration for the design of IR nanocomposites.

Despite the wide range of applications of IR nanocomposites, synthetic methodologies, characterization techniques, and the design of new reinforcements remain an object of study. In fact, the principal task in the nanocomposites obtention is by far the improvement of the efficient dispersion of the reinforcement (fibers, nanotubes, particles, whiskers, among others) in the polyisoprene matrix, wherewith homogeneous mechanical, thermal, electrical, and barrier properties in the nanocomposites will be obtained. 

Although the solid development of IR nanocomposites and their application and commercialization at the industrial level is slowed down by their high production costs, the relevance of their applications makes them the object of study in many types of research around the world with the aim of improving the cost–benefit ratio.

## Data Availability

Not applicable.

## References

[1] Ikeda Y., Kato A., Kohjiya S., Nakajima Y. (2018). Rubber Science.

[2] Kaita S., Doi Y., Kaneko K., Horiuchi A.C., Wakatsuki Y. (2004). An Efficient Gadolinium Metallocene-Based Catalyst for the Synthesis of Isoprene Rubber with Perfect 1,4-Cis Microstructure and Marked Reactivity Difference between Lanthanide Metallocenes toward Dienes As Probed by Butadiene−Isoprene Copolymerization Catalysis. Macromolecules.

[3] Gent A.N., Kawahara S., Zhao J. (1998). Crystallization and Strength of Natural Rubber and Synthetic cis-1,4-Polyisoprene. Rubber Chem. Technol..

[4] Kawahara S., Nishioka H., Yamano M., Yamamoto Y. (2022). Synthetic Rubber with the Tensile Strength of Natural Rubber. ACS Appl. Polym. Mater..

[5] Kawahara S., Chaikumpollert O., Akabori K., Yamamoto Y. (2010). Morphology and properties of natural rubber with nanomatrix of non-rubber components. Polym. Adv. Technol..

[6] Hosler D., Burkett S.L., Tarkanian M.J. (1999). Prehistoric Polymers: Rubber Processing in Ancient Mesoamerica. Science.

[7] Bode H.B., Kerkhoff K., Jendrossek D. (2001). Bacterial Degradation of Natural and Synthetic Rubber. Biomacromolecules.

[8] Rose K., Steinbuchel A. (2005). Biodegradation of Natural Rubber and Related Compounds: Recent Insights into a Hardly Understood Catabolic Capability of Microorganisms. Appl. Environ. Microbiol..

[9] Reyes-Gómez S., Montiel R., Tlenkopatchev M.A. (2018). Chicle Gum from sapodilla (Manilkara zapota) as a Renewable Resource for Metathesis Transformations. J. Mex. Chem. Soc..

[10] Bhowmick A.K., Stephens H. (2000). Handbook of Elastomers.

[11] Cornish K. (1996). Hypoallergenic Natural Rubber Products from Parthenum Argentatum (Gray) and Other Non-Hevea Brasiliensis Species. United States Patent.

[12] Schoenberg E., Marsh H.A., Walters S.J., Saltman W.M. (1979). Polyisoprene. Rubber Chem. Technol..

[13] Ricci G., Leone G., Boglia A., Boccia A.C., Zetta L. (2009). *cis*-1,4-*alt*-3,4 Polyisoprene: Synthesis and Characterization. Macromolecules.

[14] Van Amerongen G.J., Johnson B.L., Goodman M. (1966). Transition Metal Catalyst Systems for Polymerizing Butadiene and Isoprene. Elastomer Stereospecific Polymerization.

[15] Polybutadiene Rubber (BR) Product Details | Goodyear Chemical. https://www.goodyearchemical.com/products/polybutadiene-rubber.

[16] Stavely F.W., Biddison P.H., Forster M.J., Dawson H.G., Binder J.L. (1961). The Structure of Various Natural Rubbers. Rubber Chem. Technol..

[17] Thiele S.K.-H., Wilson D.R. (2003). Alternate Transition Metal Complex Based Diene Polymerization. J. Macromol. Sci. Part C Polym. Rev..

[18] Osakada K., Takeuchi D. (2004). Coordination Polymerization of Dienes, Allenes, and Methylenecycloalkanes. Polymer Synthesis. Advances in Polymer Science.

[19] Fischbach A., Meermann C., Eickerling G., Scherer W., Anwander R. (2006). Discrete Lanthanide Aryl(alk)oxide Trimethylaluminum Adducts as Isoprene Polymerization Catalysts. Macromolecules.

[20] Song J.-S., Huang B.-C., Yu D.-S. (2001). Progress of synthesis and application oftrans-1,4-polyisoprene. J. Appl. Polym. Sci..

[21] Natta G., Porri L., Giorgio M. (1955). Crystalline Linear High Polymers of Diolefins. Italy Patent.

[22] Ricci G., Italia S., Porri L. (1994). Polymerization of 1,3-dienes with methylaluminoxanetriacetylacetonatovanadium. Macromol. Chem. Phys..

[23] Bazzini C., Giarrusso A., Porri L., Pirozzi B., Napolitano R. (2004). Synthesis and characterization of syndiotactic 3,4-polyisoprene prepared with diethylbis(2,2′-bipyridine)iron–MAO. Polymer.

[24] Bazzini C., Giarrusso A., Porri L. (2002). Diethylbis(2,2′-bipyridine)iron/MAO. A Very Active and Stereospecific Catalyst for 1,3-Diene Polymerization. Macromol. Rapid Commun..

[25] Ricci G., Morganti D., Sommazzi A., Santi R., Masi F. (2003). Polymerization of 1,3-dienes with iron complexes based catalysts Influence of the ligand on catalyst activity and stereospecificity. J. Mol. Catal. A Chem..

[26] Wang B., Cui D., Lv K. (2008). Highly 3,4-Selective Living Polymerization of Isoprene with Rare Earth Metal Fluorenyl N-Heterocyclic Carbene Precursors. Macromolecules.

[27] Zhang L., Luo Y., Hou Z. (2005). Unprecedented Isospecific 3,4-Polymerization of Isoprene by Cationic Rare Earth Metal Alkyl Species Resulting from a Binuclear Precursor. J. Am. Chem. Soc..

[28] Ricci G., Battistella M., Porri L. (2001). Chemoselectivity and Stereospecificity of Chromium(II) Catalysts for 1,3-Diene Polymerization. Macromolecules.

[29] Kuzma L.J., Morton M. (1999). Polybutadiene and Polyisoprene Rubbers. Rubber Technology.

[30] Fried J.R., Prentice Hall (2014). Biopolymers, Natural Polymers, and Fibers. Polymer Science & Technology.

[31] Malaysian Rubber Council. https://www.myrubbercouncil.com/industry/world_production.php.

[32] Statista (2023). Rubber–Statistics & Facts. https://www.statista.com/topics/3268/rubber/#topicOverview.

[33] (2020). International Rubber Study Group. https://www.rubberstudy.org/welcome.

[34] Makhiyanov N., Akhmetov I.G., Vagizov A.M. (2012). Microstructure of polyisoprenes synthesized with titanium- and neodymium-containing catalytic systems. Polym. Sci. Ser. A.

[35] Rubber World Global Synthetic Rubber Market Production Forecast at 17,690 kt by 2027. https://rubberworld.com/global-synthetic-rubber-market-production-forecast-at-17690-kt-by-2027/?doing_wp_cron=1682020685.2811911106109619140625.

[36] Asghar U., Masoom A., Javed A., Abbas A. (2020). Economic Analysis of Isoprene Production from Good Year Scientific Process. Am. J. Chem. Eng..

[37] Ramos-DeValle L.F., Aramburo F. (1983). Effect of Flow-Induced Crystallization on the End Correction Factor. I. Raw Gum Elastomers. J. Rheol..

[38] Kraton Corporation, Special Polymers. https://kraton.com/.

[39] Americas International, Rubber Chemical Products. https://americasinternational.com/products-suppliers/.

[40] Zeon Corporation IR (Polyisoprene Rubber). https://www.zeon.co.jp/en/business/enterprise/rubber/ir/.

[41] Versalis Eni, Elastomers. https://www.versalis.eni.com/en-IT/portfolio/polymers-and-intermediates/elastomers.html.

[42] Sinopec Corporation, Special Rubbers. http://www.sinopecgroup.com/group/en/products/Finechem/Product/SpecialRubber.shtml.

[43] Rimpex Rubber-TPI, Trans Isoprene Rubber. http://www.rubberimpex.com/TPI/.

[44] Gutiérrez S., Tlenkopatchev M.A. (2010). Metathesis of renewable products: Degradation of natural rubber via cross-metathesis with β-pinene using Ru-alkylidene catalysts. Polym. Bull..

[45] Burelo M., Martínez A., Cruz-Morales J.A., Tlenkopatchev M.A., Gutiérrez S. (2019). Metathesis reaction from bio-based resources: Synthesis of diols and macrodiols using fatty alcohols, β-citronellol and natural rubber. Polym. Degrad. Stab..

[46] Martínez A., Tlenkopatchev M.A., Gutiérrez S., Burelo M., Vargas J., Jiménez-Regalado E. (2019). Synthesis of Unsaturated Esters by Cross-Metathesis of Terpenes and Natural Rubber Using Ru-Alkylidene Catalysts. Curr. Org. Chem..

[47] Pineda-Contreras A., Vargas J., Santiago A.A., Martínez A., Cruz-Morales J.A., Reyes-Gómez S.E., Burelo M., Gutiérrez S. (2018). Metátesis de olefinas en México: Desarrollo y aplicaciones en nuevos materiales poliméricos y en química sustentable. Mater. Av..

[48] Jain A.K., Deval R., Ramesh V., Prasad G. (2008). Natural rubber latex allergy. Indian J. Dermatol. Venereol. Leprol..

[49] Rahimi A., Mashak A. (2013). Review on rubbers in medicine: Natural, silicone and polyurethane rubbers. Plast. Rubber Compos..

[50] Kahn H., Horne S.E.J. (1965). Method of Polymerizing Butadiene-1,3-Hydrocarbons. U.S. Patent.

[51] Horne S.E., Kiehl J.P., Shipman J.J., Folt V.L., Gibbs C.F., Willson E.A., Newton E.B., Reinhart M.A. (1956). Ameripol SN—A Cis-,4-Polyisoprene. Ind. Eng. Chem..

[52] Stavely F.W. (1956). Coral Rubber—A Cis-1,4-Polyisoprene. Ind. Eng. Chem..

[53] Wakefield L.B., Foster F.C. (1972). Essentially cis Rubbery Polyisoprene and Method for Making Same. United States Patent.

[54] Senyek M.L., Herman M.F. (2008). Isoprene Polymers. Encyclopedia of Polymer Science and Technology.

[55] Ceausescu E. (1983). Stereospecific Polymerization of Isoprene.

[56] Natta G., Porri L., Mazzei A., Morero D., Natta G., Danusso F. (1967). Stereospecific polymerization of conjugated diolefins. note III: The polymerization of butadiene with the Al(C_2_H_5_)_3_-TiCl_4_ catalyst system. Stereoregular Polymers and Stereospecific Polymerizations.

[57] Natta G., Mazzanti G., Pregaglia G., Natta G., Danusso F. (1967). Organometallic complexes obtained by the reduction of hydrocarbon solutions of titanium halides with aluminum. Stereoregular Polymers and Stereospecific Polymerizations.

[58] Adams H.E., Stearns R.S., Smith W.A., Binder J.L. (1958). cis-1,4-Polyisoprene Prepared with Alkyl Aluminum and Titanium Tetrachloride. Ind. Eng. Chem..

[59] Schoenberg E., Chalfant D.L., Mayor R.H. (1964). Preformed Aluminum Triisobutyl-Titanium Tetrachloride Catalysts for Isoprene Polymerization. Rubber Chem. Technol..

[60] Morton M. (2009). Elastomers, Synthetic, Survey.

[61] Jiang B., Weng Y., Zhang S., Zhang Z., Fu Z., Fan Z. (2018). Kinetics and mechanism of ethylene polymerization with TiCl_4_/MgCl_2_ model catalysts: Effects of titanium content. J. Catal..

[62] Lovering E.G., Wright W.B. (1968). Evidence for several species of active sites in Ziegler-Natta catalysts. J. Polym. Sci. Part A-1 Polym. Chem..

[63] Ouyang J., Wang F., Shen Z. (1981). Proceedings of China-U.S. Bilateral Symposium on Polymer Chemistry and Physics.

[64] Zhiquan S., Jun O., Fusong W., Zhenya H., Fusheng Y., Baogong Q. (1980). The characteristics of lanthanide coordination catalysts and the cis-polydienes prepared therewith. J. Polym. Sci. Polym. Chem. Ed..

[65] Meyer K.H. (1950). Natural and Synthetic High Polymers.

[66] Beaman R.G. (1948). Anionic Chain Polymerization. J. Am. Chem. Soc..

[67] Overberger C.G., Pearce E.M., Mayes N. (1958). Polymerization of methacrylonitrile with lithium. J. Polym. Sci..

[68] Tobolsky A.V., Rogers C.E. (1959). Isoprene polymerization by organometallic compounds. II. J. Polym. Sci..

[69] Higginson W.C.E., Wooding N.S. (1952). 138. Anionic polymerisation. Part I. The polymerisation of styrene in liquid ammonia solution catalysed by potassium amide. J. Chem. Soc..

[70] Minoura Y., Tsubio S. (1970). Polymerization of vinyl monomers by alkali metal-thiobenzophenone complexes. J. Polym. Sci. Part A-1 Polym. Chem..

[71] Glukhovskoi V., Litvin Y., Gainulin I. (1975). The synthesis and catalytic activity of polymeric compounds containing alkali metals. Polym. Sci. USSR.

[72] Alev S., Schué F., Kaempf B. (1975). Use of dicyclohexyl-18-crown-6 in anionic polymerization. I. Solutions of alkali metals in benzene and in tetrahydrofuran. J. Polym. Sci. Polym. Lett. Ed..

[73] Hansley V.L., Greenberg H. (1965). Control of Alfin Rubber Molecular Weight. Rubber Chem. Technol..

[74] Astruc D. (2007). Organometallic Chemistry and Catalysis.

[75] Kaita S., Hou Z., Wakatsuki Y. (1999). Stereospecific Polymerization of 1,3-Butadiene with Samarocene-Based Catalysts. Macromolecules.

[76] Kaita S., Hou Z., Nishiura M., Doi Y., Kurazumi J., Horiuchi A.C., Wakatsuki Y. (2003). Ultimately Specific 1,4-cis Polymerization of 1,3-Butadiene with a Novel Gadolinium Catalyst. Macromol. Rapid Commun..

[77] (2022). Nanocomposites, Nanoparticles, Nanoclays and Nanotubes: Global Markets to 2022. https://www.bccresearch.com/market-research/nanotechnology/nanocomposites-nanoparticles-nanoclays-and-nanotubes-global-markets.html.

[78] Safdari M., Al-Haik M.S., Ismail A., Goh S.P. (2018). A Review on Polymeric Nanocomposites: Effect of Hybridization and Synergy on Electrical Properties. Carbon-Based Polymer Nanocomposites for Environmental and Energy Applications.

[79] Dong P., Prasanth R., Xu F., Wang X., Li B., Shankar R. (2015). Eco-friendly Polymer Nanocomposite, Properties and Processing. Advanced Structured Materials.

[80] Din S.H., Shah M.A., Sheikh N.A., Butt M.M. (2020). Nano-composites and their applications: A review. Charact. Appl. Nanomater..

[81] Titus D., Samuel E.J.J., Roopan S.M., Shukla A.K., Iravani S. (2019). Nanoparticle characterization techniques. Green Synthesis, Characterization and Applications of Nanoparticles.

[82] Sheiko S.S., Dobrynin A.V. (2019). Architectural Code for Rubber Elasticity: From Supersoft to Superfirm Materials. Macromolecules.

[83] Murniati R., Wibowo E., Rokhmat M., Iskandar F., Abdullah M. (2017). Natural Rubber Nanocomposite as Human-Tissue-Mimicking Materials for Replacement Cadaver in Medical Surgical Practice. Procedia Eng..

[84] Wang Z., Jiang F., Zhang Y., You Y., Wang Z., Guan Z. (2014). Bioinspired Design of Nanostructured Elastomers with Cross-Linked Soft Matrix Grafting on the Oriented Rigid Nanofibers To Mimic Mechanical Properties of Human Skin. ACS Nano.

[85] Mantilaka M.M.M.G.P.G., Wijesinghe W.P.S.L., Dissanayake D.M.S.N., Ekanayake U.G.M., Senthilnathan A., Gho L.K., Aswanthi M.K., De Silva R.T., Thomas S. (2020). Current review on the utilization of nanoparticles for ceramic matrix reinforcement. Interfaces in Particle and Fibre Reinforced Composites.

[86] Silvestre J., Silvestre N., de Brito J. (2015). An Overview on the Improvement of Mechanical Properties of Ceramics Nanocomposites. J. Nanomater..

[87] Korać M., Kamberović Ž., Anđić Z., Stopić S. (2020). Advances in Thermochemical Synthesis and Characterization of the Prepared Copper/Alumina Nanocomposites. Metals.

[88] Ghasemi M.J., Silani M., Maleki A., Jamshidian M. (2020). Micromechanical simulation and experimental investigation of aluminum-based nanocomposites. Def. Technol..

[89] Dubey A., Khosla P., Singh H.K., Katoch V., Kumar D., Gupta P. (2016). A Review on Role of Processing Parameter in Determining Properties of Silicon Carbide Reinforced Metal Matrix Nanocomposites. J. Appl. Sci. Eng..

[90] Malaki M., Xu W., Kasar A.K., Menezes P.L., Dieringa H., Varma R.S., Gupta M. (2019). Advanced Metal Matrix Nanocomposites. Metals.

[91] Usuki A., Kojima Y., Kawasumi M., Okada A., Fukushima Y., Kurauchi T., Kamigaito O. (1993). Synthesis of nylon 6-clay hybrid. J. Mater. Res..

[92] Hoque A., Ahmed M., Rahman G., Islam M., Khan M.A., Hossain M.K. (2018). Fabrication and comparative study of magnetic Fe and α-Fe2O3 nanoparticles dispersed hybrid polymer (PVA + Chitosan) novel nanocomposite film. Results Phys..

[93] Thomas S., Maria H.J. (2017). Progress in Rubber Nanocomposites.

[94] Stephen R., Thomas S., Thomas S., Stephen R. (2010). Nanocomposites: State of the Art, New Challenges and Opportunities. Rubber Nanocomposites: Preparation, Properties, and Applications.

[95] Das A., Basu D., Heinrich G., Kobayashi S., Mullen K. (2015). Rubber Nanocomposites. Encyclopedia of Polymeric Nanomaterials.

[96] Reddy K.R., Reddy C.V., Babu B., Ravindranadh K., Naveen S., Raghu A.V. (2018). Recent advances in layered clays–intercalated polymer nanohybrids: Synthesis strategies, properties, and their applications. Modified Clay and Zeolite Nanocomposite Materials.

[97] Hao L.T., Eom Y., Tran T.H., Koo J.M., Jegal J., Hwang S.Y., Oh D.X., Park J. (2019). Rediscovery of nylon upgraded by interactive biorenewable nano-fillers. Nanoscale.

[98] Martinez-Pardo I., Shanks R.A., Adhikari R., Adhikari B. (2018). Natural Rubber with Polyhedral Oligomeric Silsesquioxane, Nanocomposites, and Hybrids Compared by Molecular Modeling. Macromol. Theory Simul..

[99] Dasgupta D., Srividhya M., Sarkar A., Dubey M., Wrobel D., Saxena A. (2017). Rubber nanocomposites with polyhedral oligomeric silsesquioxanes (POSS) as the nanofiller. Progress in Rubber Nanocomposites.

[100] Zhang D., Liu Y., Shi Y., Huang G. (2013). Effect of polyhedral oligomeric silsesquioxane (POSS) on crystallization behaviors of POSS/polydimethylsiloxane rubber nanocomposites. RSC Adv..

[101] Salehiyan R., Ray S.S., Ray S.S. (2018). Rubber Nanocomposites: Processing, Structure–Property Relationships, Applications, Challenges, and Future Trends. Springer Series in Materials Science.

[102] Mohammad A., Simon G.P., Mai W.-Y., Yu Z.-Z. (2006). Rubber-clay nanocomposites. Polymer Nanocomposites.

[103] Ma J., Zhang L., Geng L., Thomas S., Stephen R. (2010). Manufacturing Techniques of Rubber Nanocomposites. Rubber Nanocomposites: Preparation, Properties, and Applications.

[104] Galimberti M. (2012). Rubber Clay Nanocomposites. Advanced Elastomers—Technology, Properties and Applications.

[105] Srivastava S.K., Mishra Y.K. (2018). Nanocarbon Reinforced Rubber Nanocomposites: Detailed Insights about Mechanical, Dynamical Mechanical Properties, Payne, and Mullin Effects. Nanomaterials.

[106] Alexandre M., Dubois P. (2000). Polymer-layered silicate nanocomposites: Preparation, properties and uses of a new class of materials. Mater. Sci. Eng. R Rep..

[107] Karger-Kocsis J., Wu C.-M. (2004). Thermoset rubber/layered silicate nanocomposites. Status and future trends. Polym. Eng. Sci..

[108] Messori M., Bignotti F., De Santis R., Taurino R. (2009). Modification of isoprene rubber by in situ silica generation. Polym. Int..

[109] Kohjiya S., Ikeda Y. (2003). In Situ Formation of Particulate Silica in Natural Rubber Matrix by the Sol-Gel Reaction. J. Sol-Gel Sci. Technol..

[110] Peng Z., Kong L.X., Li S.-D., Chen Y., Huang M.F. (2007). Self-assembled natural rubber/silica nanocomposites: Its preparation and characterization. Compos. Sci. Technol..

[111] Cao L., Sinha T.K., Zhang X., Zhai X., Wang C., Zong C., Kim J.K. (2019). Graphene/carbon nanotubes-supported Ziegler-Natta catalysts for in situ synthesis of mechanically strong, thermally and electrically conductive trans-polyisoprene nanocomposite. J. Polym. Res..

[112] Liu J., Wang Z., Li S., Teng J., Min B. (2019). Development of functionalized core–shell nanohybrid/synthetic rubber nanocomposites with enhanced performance. Soft Matter.

[113] Bokobza L. (2019). Natural Rubber Nanocomposites: A Review. Nanomaterials.

[114] Khani M.M., Abbas Z.M., Benicewicz B.C. (2017). Well-defined polyisoprene-grafted silica nanoparticles via the RAFT process. J. Polym. Sci. Part A Polym. Chem..

[115] Kongsinlark A., Rempel G.L., Prasassarakich P. (2012). Synthesis of monodispersed polyisoprene–silica nanoparticles via differential microemulsion polymerization and mechanical properties of polyisoprene nanocomposite. Chem. Eng. J..

[116] Burelo M., Gaytán I., Loza-Tavera H., Cruz-Morales J.A., Zárate-Saldaña D., Cruz-Gómez M.J., Gutiérrez S. (2022). Synthesis, characterization and biodegradation studies of polyurethanes: Effect of unsaturation on biodegradability. Chemosphere.

[117] Hamed G.R., Gent A.N. (2012). Materials and Compounds. Engineering with Rubber.

[118] Ray S.S., Okamoto M. (2003). Polymer/layered silicate nanocomposites: A review from preparation to processing. Prog. Polym. Sci..

[119] El Fray M., Goettler L.A., Thomas S., Stephen R. (2010). Application of Rubber Nanocomposites. Rubber Nanocomposites: Preparation, Properties, and Applications.

[120] Thomas S., Thomas S., Abraham J., George S.C., Thomas S. (2018). Investigation of the mechanical, thermal and transport properties of NR/NBR blends: Impact of organoclay content. J. Polym. Res..

[121] Brydson J.A. (1988). Rubbery Materials and Their Compounds.

[122] Galimberti M., Senatore S., Lostritto A., Giannini L., Conzatti L., Costa G., Guerra G. (2009). Reinforcement of diene elastomers by organically modified layered silicates. e-Polymers.

[123] Liu C., Qin H., Mather P.T. (2007). Review of progress in shape-memory polymers. J. Mater. Chem..

[124] Qi X., Zhang Y., Zhang L., Yue D. (2023). Bioinspired Sustainable Polymer with Stereochemistry-Controllable Thermomechanical Properties. Macromolecules.

[125] Niu Z., Wu R., Yang Y., Huang L., Fan W., Dai Q., Cui L., He J., Bai C. (2021). Recyclable, robust and shape memory vitrified polyisoprene composite prepared through a green methodology. Polymer.

[126] Donnet J.-B., Custodero E., Erman B., Mark J.E., Roland C.M. (2013). Reinforcement of Elastomers by Particulate Fillers. The Science and Technology of Rubber.

[127] Tsukada G., Tokuda M., Torii M. (2014). Temperature Triggered Shape Memory Effect of Transpolyisoprene-based Polymer. J. Endod..

[128] Wang Z., Su M., Duan X., Yao X., Han X., Song J., Ma L. (2022). Molecular Dynamics Simulation of the Thermomechanical and Tribological Properties of Graphene-Reinforced Natural Rubber Nanocomposites. Polymers.

[129] Sui G., Zhong W., Yang X., Yu Y. (2008). Curing kinetics and mechanical behavior of natural rubber reinforced with pretreated carbon nanotubes. Mater. Sci. Eng. A.

[130] Burelo M., Gutiérrez S., Treviño-Quintanilla C.D., Cruz-Morales J.A., Martínez A., López-Morales S. (2022). Synthesis of Biobased Hydroxyl-Terminated Oligomers by Metathesis Degradation of Industrial Rubbers SBS and PB: Tailor-Made Unsaturated Diols and Polyols. Polymers.

[131] Nah C., Kim D.H., Kim W.D., Kwacheon W.S., Kaang S. (2004). Friction and abrasion properties of in-situ silica-filled natural rubber nanocomposites using sol-gel process. Kautschuk Gummi Kunststoffe.

[132] Wu Y.-P., Ma Y., Wang Y.-Q., Zhang L.-Q. (2004). Effects of Characteristics of Rubber, Mixing and Vulcanization on the Structure and Properties of Rubber/Clay Nanocomposites by Melt Blending. Macromol. Mater. Eng..

[133] Wichaita W., Promlok D., Sudjaipraparat N., Sripraphot S., Suteewong T., Tangboriboonrat P. (2021). A concise review on design and control of structured natural rubber latex particles as engineering nanocomposites. Eur. Polym. J..

[134] Appamato I., Bunriw W., Harnchana V., Siriwong C., Mongkolthanaruk W., Thongbai P., Chanthad C., Chompoosor A., Ruangchai S., Prada T. (2022). Engineering Triboelectric Charge in Natural Rubber–Ag Nanocomposite for Enhancing Electrical Output of a Triboelectric Nanogenerator. ACS Appl. Mater. Interfaces.

[135] Arroyo M., López-Manchado M., Herrero B. (2003). Organo-montmorillonite as substitute of carbon black in natural rubber compounds. Polymer.

[136] Wang J., Chen D. (2013). Mechanical Properties of Natural Rubber Nanocomposites Filled with Thermally Treated Attapulgite. J. Nanomater..

